# Bioinformatic analysis of the membrane cofactor protein CD46 and microRNA expression in hepatocellular carcinoma

**DOI:** 10.3892/or.2013.2877

**Published:** 2013-11-28

**Authors:** ZEJUN LU, CHUANFU ZHANG, JIAJUN CUI, QI SONG, LIGUI WANG, JINGBO KANG, PENG LI, XIAOFENG HU, HONGBIN SONG, JINLIANG YANG, YANSONG SUN

**Affiliations:** 1 Institute of Disease Control and Prevention, Chinese Academy of Military Medical Sciences, Beijing 100071, P.R. China; 2 State Key Laboratory of Biotherapy and Cancer Center, West China Hospital, Sichuan University, Chengdu, Sichuan 610041, P.R. China; 3 Department of Radiation Oncology, Naval General Hospital of PLA, Beijing 100048, P.R. China; 4 Department of Gynaecology and Obstetrics, The General Hospital of the Chinese People’s Armed Police Force, Beijing 100039, P.R. China

**Keywords:** hepatocellular carcinoma, CD46, microRNA, let-7b, miR-17

## Abstract

The therapeutic potential of membrane complement regulatory protein (mCRP)-neutralizing antibodies is unsatisfactory, which perhaps lies in the complex role of mCRPs in tumor occurrence and development. As a member of the mCRPs, CD46 is a transmembrane protein with a cytoplasmic domain and is implicated more in the control of the alternative complement pathway than of the classical complement pathway. Growing evidence has revealed that both the CD46 signaling pathway and microRNAs (miRNAs) play an important role in the development and progression of hepatocellular carcinoma (HCC). In the present study, we analyzed mCRP expression in different tumor tissues by employing western blotting and qPCR. To address the potential role of miRNAs in CD46 signaling, we set out to profile miRNA expression in CD46-overexpressed and -silenced HepG2 cell lines. Furthermore, bioinformatic analysis was performed to identify downstream targets of CD46 signaling. We found that the levels of CD46 expression in HCC tissues were significantly higher compared to that in the adjacent normal tissues. After complement-related gene expression profiling and unsupervised hierarchical clustering analysis of 10 HCC tissues, a total of 37 miRNAs showed significantly different expression levels before and after CD46 expression change. By bioinformatic analysis, we identified let-7b and miR-17 as downstream targets of CD46 signaling, and that the expression levels of let-7b and miR-17 were negatively correlated with that of CD46 in HepG2 cells. The present study suggests that CD46 plays an important role in HCC carcinogenesis by regulating let-7b and miR-17.

## Introduction

Hepatocellular carcinoma (HCC) is the fifth most common cancer worldwide and the third most common cause of death from cancer. HCC results in more than 600,000 deaths each year (1). Surgical intervention is the main treatment for HCC. However, no more than 20% of patients with HCC are indicated to undergo surgery procedures. In addition, chemotherapy and radiotherapy have limited efficacy for the majority of HCC patients at an advanced stage (2). Thus, it is of importance to seek cancer-specific therapeutic targets and develop effective alternative approaches to specifically treat HCC.

The complement system is a set of biochemical pathways that removes pathogen components from an organism as part of the innate and acquired immune systems. Activation of the complement system triggers a wide range of cellular responses ranging from apoptosis to opsonization (3). To prevent self damage of the overactivation of the complement system, host cells express several membrane complement regulatory proteins (mCRPs) that can inhibit complement activation (4). The group of mCRPs consists of CD35 [complement receptor 1 (CR1)], CD46 (membrane cofactor protein), CD55 (decay-accelerating factor) and CD59 (protectin) (5).

As a member of the mCRPs, membrane cofactor protein (MCP) CD46 acts as a cofactor for the cleavage of C3b and C4b by the serum protease factor I (6). Moreover, CD46 also acts as a costimulatory factor for T cells along with CD3 and IL-2 *in vitro* which induces the differentiation of CD4^+^ cells into T-regulatory cells (7).

The liver is responsible for biosynthesis of approximately 80–90% of plasma complement components and expresses a variety of complement receptors. It has been widely recognized that serum complement levels are different in patients with various forms of malignancies including HCC (8). mCRPs provide protection against the attack of the homologous complement, which is necessary for *in vivo* hepatoma growth (9).

Recent evidence suggests that MCP expression in HCC is significantly higher than that in both liver cirrhosis and chronic hepatitis, which may cause HCC cells to escape from tumor-specific complement-mediated cytotoxicity (10). In addition, CD46 expression was found to be increased following addition of IL-1β and decreased upon treatment with interferon-γ (11). Although the roles of CD46 in complement activation have recently been postulated, their pathophysiological contributions to HCC are still largely unknown. Understanding the role of CD46 in HCC development is important for the development of effective means of prevention and treatment of this highly malignant form of cancer.

Recent studies have indicated that microRNAs (miRNAs) play important roles in HCC development (12,13). The expression of various miRNAs is deregulated in human HCC in comparison with matched non-neoplastic tissue (14). In some cases, aberrantly expressed miRNAs may be linked to cancer-associated pathways, indicating a direct role in liver tumorigenesis.

Here, we confirmed that the levels of CD46 expression in HCC tissues were significantly higher than that in the adjacent normal tissues. Then, we aimed to identify key miRNAs involved in CD46-related pathways based on the expression patterns in CD46-altered HepG2 cell lines. The present study represents a basic model that offers insight into the effects of cellular miRNAs and complement regulatory protein CD46 during HCC pathogenesis.

## Materials and methods

### Clinical specimens

Fresh HCC tissues and the adjacent normal tissues for microarray analysis were obtained from 10 patients who underwent surgical resection at the Navy General Hospital (Beijing, China). Histodifferentiation grading of specimens was carried out according to Edmondson-Steiner grading by experienced pathologists. Surgical pathological staging was carried out according to the modified UICC classification (15). A summary of the detailed clinicopathological information for HCC patients is shown in Table I. Paraffin-embedded liver, breast, lung, kidney and colon cancer tissues, and the adjacent normal tissues were also obtained from the Navy General Hospital. Informed consent was obtained from all of the patients or their relatives prior to analysis, and the project was approved by the Institutional Ethics Committee of the Navy General Hospital.

### Cell culture

Human hepatoma cell line HepG2 was purchased from the American Type Culture Collection (ATCC, Rockville, MD, USA) and maintained in Dulbecco’s modified Eagle’s medium (DMEM; Gibco-BRL, USA) supplemented with 10% fetal calf serum (HyClone, USA), penicillin (10^7^ U/l) and streptomycin (10 mg/l) at 37°C in a 5% CO_2_ atmosphere.

### Transient transfection of the CD46 expression plasmid

Construction of the CD46 expression plasmid and CD46 short interfering RNA (siRNA) was carried out. The cDNA fragment encoding CD46 was purchased from the Proteintech Group, Inc. (BC030594) and then cloned into the pcDNA3.1/myc-His C expression vector (Invitrogen Life Technologies, USA) between the *Hind*III and *Xho*I restriction sites. The cloned cDNA was verified by sequencing.

HepG2 cells were plated at 3×10^5^/well in 6-well plates and incubated until they reached 95% confluency. Cells were then transiently transfected with Lipofectamine 2000 (Invitrogen Life Technologies) according to the manufacturer’s recommendations. Plasmid DNA (4.0 μg) and 10 μl of Lipofectamine 2000 were diluted separately in DMEM and incubated for 5 min. They were then combined and incubated for 30 min at room temperature. The complexes were added to each well and mixed gently, followed by incubation at 37°C. Six hours later, the medium was replaced with DMEM containing 10% fetal bovine serum. Cells were then incubated for 48 and 72 h, respectively, for RNA isolation and protein extraction.

### siRNA transfection

CD46 siRNA and negative control siRNA were synthesized using phosphorothioate chemistry by Biomics (Nantong, China). HepG2 cells were plated at 1×10^5^/well in 6-well plates and incubated until they reached 50% confluency. Cells were transfected with CD46 siRNA or the negative control siRNA at a final concentration of 50 nM with Lipofectamine 2000 according to the manufacturer’s instructions. Six hours after initiation of transfection, cells were starved in serum-free DMEM for another 6 h, followed by replacement with DMEM containing 10% fetal bovine serum.

### RNA isolation and quantitative reverse transcription-PCR (qRT-PCR)

Total RNAs were isolated using the RNeasy Mini kit (Qiagen, Valencia, CA, USA) from 4 independent biological replicates according to the manufacturer’s instructions. First-strand cDNA was reversely transcribed from 1 μg total RNA in a final volume of 20 μl using RTase and random hexamers from the ExScript™ Reagent kit (Takara Bio, Inc., Dalian, China) according to the manufacturer’s instructions. Primers were designed mainly using Primer Premier 5 software, and a database search using NCBI BLAST program was performed to ensure specificity. The primer sequences are listed in Table II. PCR was performed with rTaq (Takara Bio, Inc.) in a gradient DNA thermal cycler (Bio-Rad) according to a touchdown protocol as follows: 1 cycle of 95°C for 3 min; 10 cycles of 94°C for 45 sec, annealing for 45 sec (the annealing temperature was set to 5°C above the expected annealing temperature with a decrease of 1°C for every cycle), 72°C for 1 min; 25 additional cycles with the expected annealing temperature; a final extension at 72°C for 10 min and holding at 4°C. The amount of cDNA used for each PCR reaction was 20 ng in a 25 μl reaction volume. The PCR products (5 μl) were analyzed by electrophoresis on 2% agarose gels and visualized by SYBR Gold (Molecular Probes, Eugene, OR, USA) staining.

### Western blot analysis

Western blot analysis was performed as described previously (16). Briefly, 30 μg of proteins was separated by 12% SDS-PAGE and transferred to PVDF membranes (Millipore). After incubation in blocking buffer (1X TBS, 0.1% Tween-20 and 5% w/v dry nonfat milk) for 1 h at room temperature, membranes were incubated with the antibodies. Then the membrane was incubated with the secondary antibodies for 45 min at room temperature. Reactive bands were detected using enhanced chemiluminescence (Amersham Biosciences Corp., Piscataway, NJ, USA).

### miRNA microarray analysis

Human miRNA microarray was performed by Phalanx Biotech (Hsinchu, Taiwan). The total RNA sample (2.5 μg) was size fractionated using a 100K Nanosep (Pall Corporation, USA) and small RNAs (<300 nt) were labeled with Cy5 NHS Ester fluorescence dye (GE Amersham, USA) using miRNA ULS™ Labeling kit (Kreatech Diagnostics, The Netherlands). Hybridization was executed overnight at 37°C on a Human miRNA OneArray^®^ v4.0 with Phalanx hybridization buffer using OneArray^®^ hybridization chamber (17). On the chip, each unique probe which was spotted in triplicate format, consisted of a chemically modified nucleotide-coding segment complementary to target miRNA (1,884 human miRNA entries, miRBase version 18.0; http://microrna.sanger.ac.uk/sequences/) and a spacer segment to extend the coding segment away from the substrate. Hybridization melting temperatures were balanced by modifications of the detection probes. Hybridization images were collected using a laser scanner (GenePix 4000B) and digitized using GenePix 4.1 software (both from Molecular Devices, Sunnyvale, CA, USA).

### Description of the in depth analysis

Multiple sample analysis included normalization, data adjustment, t-test/ANOVA analysis and clustering. Normalization was carried out by the 75% media scaling method by R 2.12.1. on the background-subtracted data. The normalization was performed to remove system-related variations, such as sample amount variations, different labeling dyes and signal gain differences of scanners so that biological variations were revealed. Data adjustment included data filtering, log2 transformation, gene centering and normalization. Data filtering removed miRNAs which flags were −50 across all samples. The log2 transformation converted intensity values into log2 scale. Gene centering and normalization transformed the log2 values using the mean and the standard deviation of individual genes across all samples using the following formula: Value = [(value) − mean (gene)]/[(standard deviation (gene)]. The t-test was performed between ‘control’ and ‘test’ sample groups with each group containing at least 2 samples (18). T-values were calculated for each miRNA, and p-values were computed from the theoretical t-distribution. The hierarchical clustering was carried out by Cluster 3.0 software with miRNA probes with a difference between the maximum and minimum intensity values exceeding 100 among all microarrays. The clustering plot was generated using TreeView software from Stanford University.

## Results

### Expression of CD46 in the cancer and the adjacent normal tissues

**Figure 1:**
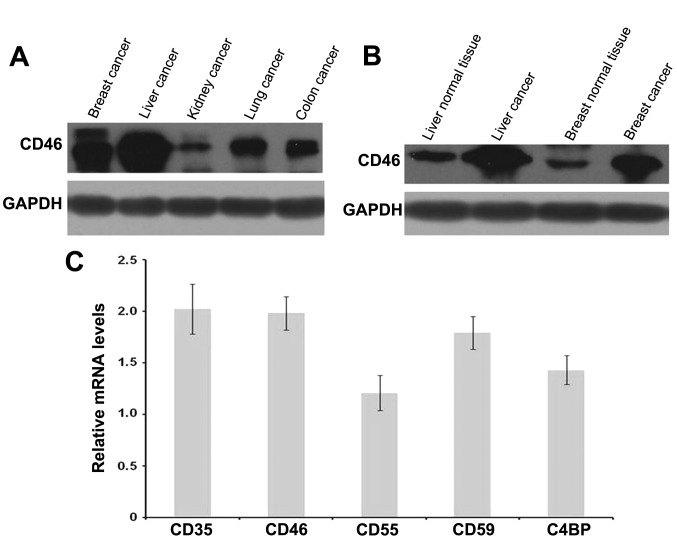
Western blotting and qRT-PCR analysis of mCRP expression in different tumor tissues. (A) CD46 expression in different types of tumor tissues: breast, liver, lung, kidney and colon cancer. (B) Expression of CD46 in liver and breast cancer as well as the adjacent normal tissues. (C) qRT-PCR analysis of the expression of CD35, CD46, CD55, CD59, C4BP. Levels of the indicated mRNAs in HepG2 cells are shown relative to the level of GAPDH mRNA. Values are expressed as means ± SD. GAPDH was used for normalization both in western blotting and qRT-PCR analysis. mCRP, membrane complement regulatory protein.

Western blotting revealed that CD46 was expressed in liver, breast, lung, kidney and colon cancer as well as the adjacent normal tissues (Fig. 1A). The levels of CD46 expression in HCC tissues were significantly higher than those in the other tumor tissues and the adjacent normal tissues (Fig. 1B). mRNA levels of CD35, CD46, CD55, CD59, C4BP were determined by quantitative real-time RT-PCR (Fig. 1C).

### Expression of CD46 in pcDNA3.1-CD46 and CD46 siRNA-transfected HepG2 cell lines

**Figure 2:**
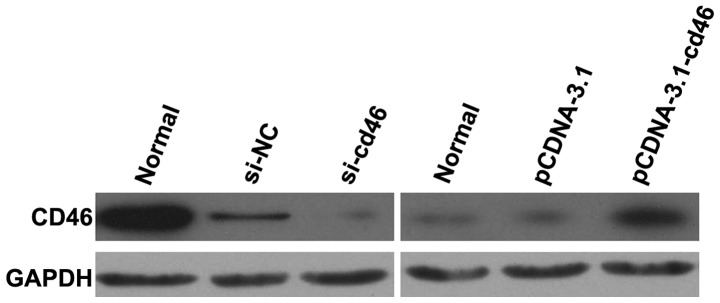
Western blot analysis of CD46 in HepG2 cell lines transfected with the different plasmids. From left to right: HepG2 cells without plasmid transfection, HepG2 cells transfected with negative control siRNA, HepG2 cells transfected with si-cd46, HGep2 cells without plasmid transfection, HepG2 cells transfected with pcDNA3.1-CD46, HepG2 cells transfected with pcDNA3.1. GAPDH (lower panel) was used as reference. siRNA, short interfering RNA.

HepG2 cells were transfected with pcDNA3.1, pcDNA3.1-CD46, CD46 siRNA or the negative control siRNA. The levels of CD46 protein were then determined in each of the cell lines by western blotting (Fig. 2). The levels of CD46 expression were significantly higher in the HepG2-pcDNA3.1-CD46 cells when compared to the levels of CD46 expression in the HepG2 and HepG2-pcDNA3.1 cells. In addition, western blotting demonstrated that CD46 protein expression was downregulated significantly at 48 h following CD46 siRNA treatment.

### Microarray analysis of the complement-related gene expression profile

**Figure 3:**
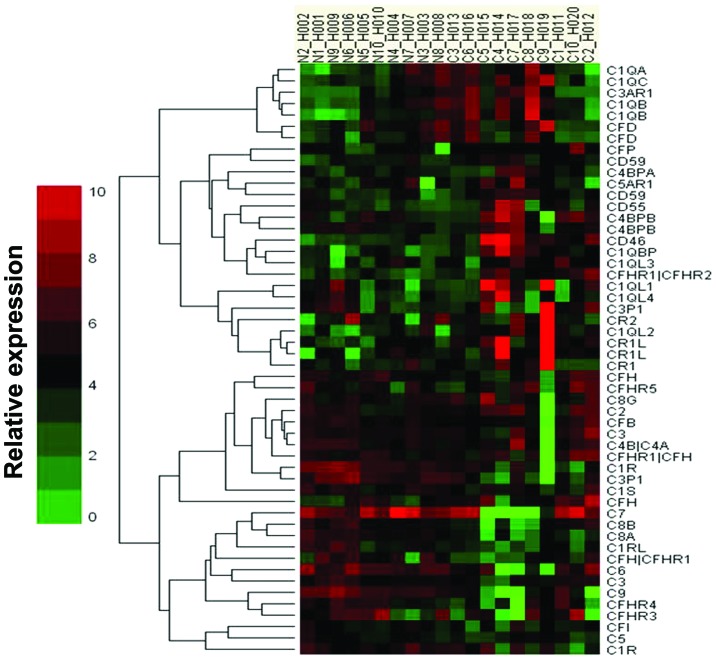
Complement-related gene expression profiling and unsupervised hierarchical clustering analysis of 10 HCC tissues. Each row represents the relative levels of expression for a single gene and each column shows the expression levels for a single sample. The red or green color indicates relatively high or low expression, respectively. HCC, hepatocellular carcinoma.

We initially screened miRNA expression in HCC tissues and the adjacent normal tissues from 10 patients, using a microarray platform that covered a total of 875 human miRNAs (Sanger miRBase release 13.0). Our analysis was limited to the set of genes encoding complement-related proteins differentially expressed between normal tissues and HCC tissues. Twenty-three miRNAs exhibited a downregulated trend and 22 exhibited an upregulating trend. Among these, CD46 was the most significantly upregulated in the HCC tissues (Fig. 3).

**Figure 4:**
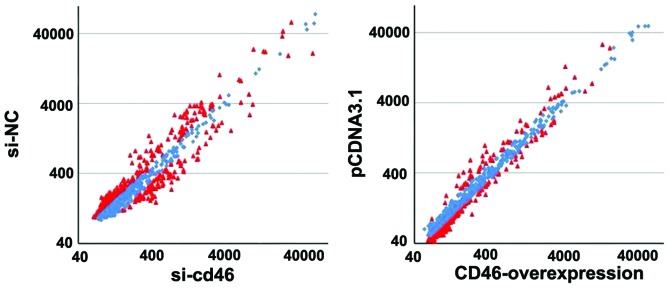
Scatter plot showing expression of miRNAs in HepG2 cells transfected with si-CD46 and pcDNA3.1-CD46. Green points indicate the 1.5-fold threshold. Red points indicate the differentially expressed miRNAs.

To explore the potential association between CD46 and miRNAs, we first performed miRNA expression profiling following CD46 overexpression using the miRNA array. Our profiling analysis revealed 293 miRNAs to be differentially expressed in HepG2 cells transfected with si-CD46 and si-NC, while 82 miRNAs were differentially expressed in HepG2 cells transfected with pcDNA3.1 and pcDNA3.1-CD46 (Fig. 4). Through comparison of the miRNA levels in both groups, we found that 37 miRNAs were inverse expressed before and after CD46 expression change (P<0.05; Table III).

### Bioinformatic analysis

**Figure 5:**
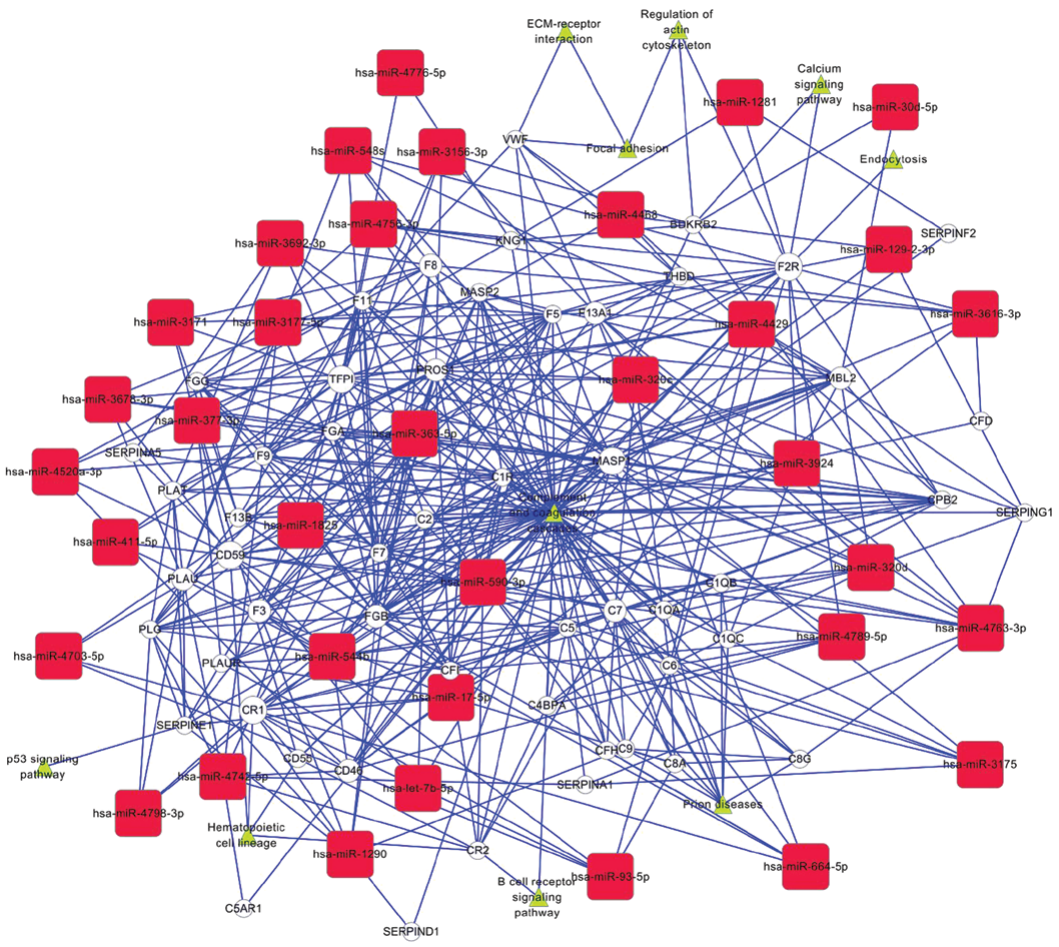
Presumed connections between validated miRNAs and complement-related genes. Genes are represented by circles. Differentially expressed miRNAs are represented by boxes. Triangles represent a Kegg pathway. Lines represent interactions.

To better understand how the functions of these experimentally validated miRNAs and the complement-related genes (Table III) are involved in the neoplastic progression of HCC, we performed a gene interaction network analysis (Fig. 5). The CD46-related network of miRNA-mRNA interaction, representing the critical miRNAs and their targets, was established according to the miRNA degree. Our results revealed that miRNAs regulated by CD46 were hsa-let-7b, hsa-miR-17 and hsa-miR-590-3p. We confirmed the result by the miRNA target prediction web tools of DIANA-microT (version 3.0, www.microrna.gr/microT) and TargetScan (release 6.2, http://www.targetscan.org/). In addition, let-7b, miR-17-5p, miR-590-3p were also found to have direct or indirect association with other members of the complement family, such as CR1, CD55 and CD59.

## Discussion

It is likely that the therapeutic potential of monoclonal antibodies such as rituximab and trastuzumab, is largely impaired by the mCRPs (19). It is important to note that the number of reports demonstrating increased expression of mCRPs in tumors relative to the corresponding normal tissue is consistently increasing (20).

For example, the expression of CD46 in primary cervix tissue was found to increase from normal to premalignant to malignant (21). Here, we also found that the levels of CD46 expression in HCC tissues were significantly higher than those in other types of tumor tissues and in the adjacent normal tissues. For cancer therapy, the aim should be to identify a treatment that reduces mCRP expression.

However, due to the fact that even after the blocking of mCRPs, some tumors still remain resistant to complement-mediated lysis and that mCRP expression *in vivo* in cancers is largely heterogeneous with specimens lacking or having low mCRP expression, the expectations from mCRP-targeted therapies are slightly low (22,23). Why did this paradox occur? The reason for this may be that previous studies only focused on the inhibitive effect of mCRP on the complement-dependent cellular cytotoxicity (CDCC), but ignored the role of mCRP in tumor occurrence and development. Thus, the role of mCRP in tumor development must be exploited.

In contrast to CD55 and CD59, both CD35 and CD46 are transmembrane proteins with a cytoplasmatic domain. CD46 is implicated more in the control of the alternative complement pathway than of the classical complement pathway (24). Studies have shown that expression of CD46 was decreased in hepatoma cells upon treatment with interferon-γ (11).

The efficacy of antisense technology to specifically knockdown the expression of selected CRPs in tumors has been demonstrated (25). Having identified several miRNAs that may be of value for the understanding of hepatoma development, here, we set out to compare miRNA expression patterns in CD46-overexpressed and -silenced HepG2 cell lines by applying microarray and qPCR profiling techniques. In the present study, we identified 37 miRNAs with significantly differential expression levels before and after CD46 expression change.

To link CD46-regulated miRNA profiling data with biological consequences, we used a computational approach to examine the possible pathways collectively regulated by the downregulation or upregulation of miRNAs on the basis of their predicted targets. In addition, inverse correlation of miRNA expression with their tentative target genes is a useful approach to reduce the generally extensive number of computationally predicted target genes for further analysis. Here, network analysis results revealed that CD46-regulated miRNAs included let-7b and miR-17-5p.

Many studies have shown that miRNAs are implicated in many types of cancers, and altered miRNA levels can result in the aberrant expression of gene products that may contribute to cancer biology (26). Moreover, certain miRNAs have been classified as tumor-suppressors or oncogenes (27). The let-7 miRNA is conserved in invertebrates and vertebrates, and was originally discovered in the nematode *Caenorhabditis elegans*, where it regulates cell proliferation and differentiation (28). Increasing evidence has revealed that let-7 is deregulated in various types of cancer cells, such as colorectal, lung, colon, ovarian, breast and gastric cancer (29). Recently, it has been reported that members of the let-7 family are downregulated in HCC carcinoma (30). Overexpression of let-7 in liver cancer cell lines alters cell cycle progression and reduces cell division, providing evidence that let-7 functions as a tumor-suppressor in HCC.

Additionally, as a key oncogenic component of miR-17-92, miR-17-5p was supposed to be regulated by CD46 in HepG2 cell lines. The association of miR-17-92 with a broad range of cancers not only underlines the clinical significance of this locus but also suggests that miR-17-92 may regulate fundamental biological processes. miR-17-5p was found to be overexpressed in HCC (31). Some targets of miR-17-5p have been confirmed, such as E2F1, NCOA3 and HSP27 (32). Furthermore, recent research has demonstrated that the level of miR-17-5p is associated with development of HCC and can serve as a non-invasive biomarker for the prognostic prediction of HCC patients (33,34).

In the present study, to identify clusters of miRNAs and pathways directly or indirectly regulated by CD46, a microarray and bioinformatic analysis approach was employed to compare the proteome of HepG2 cells transfected with pcDNA3.1-CD46 and CD46 siRNA. To our knowledge, the present study was the first to take a broad-based approach to identify the downstream effects of membrane cofactor protein (MCP) CD46. The study provides functional data linking CD46 to oncogenic characteristics of HCC. However, many regulated spots remain unidentified in this complicated network. Elucidation of all the physiological changes and the mechanisms responsible for CRPs requires further research.

## Figures and Tables

**Table I:** Clinicopathological features of the hepatocellular carcinoma samples.

Clinicopathological features	No.	Mean patient age (years)
Hepatocellular carcinoma	10	56.6
Adjacent normal tissues	10	56.6
Histodifferentiation grade		
Well differentiated	2	58
Moderately differentiated	6	56.3
Poorly differentiated	2	56
Surgical pathological stage		
I	1	61
II	7	56.1
III	2	56

**Table II:** Sequences of the forward and reverse primers of complement regulatory genes.

Primer name		Sequence
CD35	CD35-F	TAGATGTGCTTGGGGAGAATGGGG
	CD35-R	AGACGAGGAACCAATGAGTCGG
C4BP	C4BP-F	GAGTGCCGCTTGGGCCACTGTCCT
	C4BP-R	CAGAGGTTCTTACTCTCCTGAAAGGCAAG
CD55	CD55-R	CCTTATCACCATCAACACCCCTGG
	CD55-F	AGGCATTTTCATCTTTCCTTCGGG
CD46	CD46-R	CTAGGACCTGAGGCACTGGACG
	CD46-F	CCAAAGTGTCTTAAAGTGCTGCCTC
CD59	CD59-F	GAGCCCAGGGAGGGAAAGGTTC
	CD59-R	CGAGGTTAAGGCAAAACCCTACGG

**Table III:** microRNA array analysis reveals 37 miRNAs responsive to CD46 expression change in HepG2 cells (P<0.01).

	Normalized intensity				Log ratio		P-value	
			
microRNA name	si-CD46	si-NC	pcDNA3.1-CD46	pcDNA3.1	pcDNA3.1-CD46/pcDNA3.1	si-NC/si-CD46	pcDNA3.1-CD46/pcDNA3.1	si-NC/si-CD46
hsa-let-7b	1,739.90	932.24	476.43	738.06	−0.63	−0.90	0.00178	0.00063
hsa-miR-1281	1,866.42	4,151.44	2,411.89	1,513.13	0.67	1.15	0.00072	0.00001
hsa-miR-129-3p	384.62	850.57	707.68	460.06	0.62	1.14	0.00060	0.00015
hsa-miR-1290	9,307.82	3,865.57	6,019.03	9,277.45	−0.62	−1.27	0.00024	0.00002
hsa-miR-17-5p	1,067.83	449.22	336.20	711.62	−1.08	−1.25	0.00001	0.00009
hsa-miR-1825	1,996.99	3,048.80	2,162.06	1,417.19	0.61	0.61	0.00056	0.00010
hsa-miR-30d	489.89	257.70	229.39	618.70	−1.43	−0.93	0.00000	0.00067
hsa-miR-3156-3p	96.16	153.50	124.45	80.83	0.62	0.67	0.00053	0.00047
hsa-miR-3171	81.98	181.66	96.59	62.70	0.62	1.15	0.00069	0.00009
hsa-miR-3175	8,277.44	5,401.94	4,485.72	13,183.03	−1.56	−0.62	0.00000	0.00002
hsa-miR-3177-5p	105.26	191.52	121.66	64.97	0.91	0.86	0.00360	0.00009
hsa-miR-320c	985.84	559.06	645.46	1,098.40	−0.77	−0.82	0.00009	0.00026
hsa-miR-320d	678.15	388.67	361.27	995.66	−1.46	−0.80	0.00003	0.00094
hsa-miR-3616-3p	97.17	161.95	96.59	62.70	0.62	0.74	0.00222	0.00082
hsa-miR-363^*^	8,478.86	2,758.71	2,635.71	6,659.13	−1.34	−1.62	0.00122	0.00014
hsa-miR-3678-3p	96.16	176.03	99.37	63.46	0.65	0.87	0.00560	0.00108
hsa-miR-3692	105.26	160.54	99.37	64.21	0.63	0.61	0.00463	0.00075
hsa-miR-377	98.18	173.21	87.30	56.66	0.62	0.82	0.00158	0.00157
hsa-miR-3924	79.96	142.23	92.87	53.64	0.79	0.83	0.00608	0.00185
hsa-miR-411	101.22	177.44	108.66	64.21	0.76	0.81	0.01089	0.00013
hsa-miR-4429	540.49	312.63	338.05	595.28	−0.82	−0.79	0.00026	0.00001
hsa-miR-4468	95.14	157.72	103.09	61.19	0.75	0.73	0.00476	0.00008
hsa-miR-4520a-3p	111.34	194.33	133.74	83.85	0.67	0.80	0.00003	0.00061
hsa-miR-4636	104.25	197.15	108.66	67.23	0.69	0.92	0.00187	0.00010
hsa-miR-4703-5p	97.17	166.17	98.44	62.70	0.65	0.77	0.00069	0.00002
hsa-miR-4742-5p	114.37	181.66	106.80	67.99	0.65	0.67	0.00114	0.00432
hsa-miR-4756-3p	107.29	171.80	109.59	67.99	0.69	0.68	0.00001	0.00025
hsa-miR-4763-3p	6,437.34	4,137.36	2,828.88	4,596.04	−0.70	−0.64	0.00392	0.00303
hsa-miR-4776-5p	571.87	346.42	314.84	500.85	−0.67	−0.72	0.00047	0.00327
hsa-miR-4783-5p	102.23	185.89	99.37	64.97	0.61	0.86	0.00871	0.00138
hsa-miR-4789-5p	84.01	143.64	81.73	52.88	0.63	0.77	0.00028	0.00135
hsa-miR-4798-3p	96.16	157.72	84.51	53.64	0.66	0.71	0.02291	0.00457
hsa-miR-544b	102.23	170.39	108.66	71.01	0.61	0.74	0.00530	0.00061
hsa-miR-548s	97.17	170.39	98.44	62.70	0.65	0.81	0.00022	0.00059
hsa-miR-590-3p	109.31	191.52	110.52	71.77	0.62	0.81	0.00134	0.00106
hsa-miR-664^*^	566.81	284.46	158.81	370.92	−1.22	−0.99	0.00020	0.00029
hsa-miR-93	791.51	356.28	304.62	468.37	−0.62	−1.15	0.00002	0.00046

## References

[1] Duffy A, Greten T (2010). Developing better treatments in hepatocellular carcinoma. Expert Rev Gastroenterol Hepatol.

[2] Lo CM, Ngan H, Tso WK (2002). Randomized controlled trial of transarterial lipiodol chemoembolization for unresectable hepatocellular carcinoma. Hepatology.

[3] Holt DS, Botto M, Bygrave AE, Hanna SM, Walport MJ, Morgan BP (2001). Targeted deletion of the CD59 gene causes spontaneous intravascular hemolysis and hemoglobinuria. Blood.

[4] Halperin JA, Taratuska A, Nicholson-Weller A (1993). Terminal complement complex C5b-9 stimulates mitogenesis in 3T3 cells. J Clin Invest.

[5] Hadders MA, Bubeck D, Roversi P (2012). Assembly and regulation of the membrane attack complex based on structures of C5b6 and sC5b9. Cell Rep.

[6] Brodbeck WG, Mold C, Atkinson JP, Medof ME (2000). Cooperation between decay-accelerating factor and in protecting cells from autologous complement attack. J Immunol.

[7] Kemper C, Chan AC, Green JM, Brett KA, Murphy KM, Atkinson JP (2003). Activation of human CD4^+^cells with CD3 and CD46 induces a T-regulatory cell 1 phenotype. Nature.

[8] Matsumura N, Tagami H, Hotta T, Takemura S, Yoshikawa T, Kondo M (1981). Serum complement profile and its clinical significance in patients with hepatocellular carcinoma and liver cirrhosis. Nihon Shokakibyo Gakkai Zasshi.

[9] Baranyi L, Baranji K, Takizawa H, Okada N, Okada H (1994). Cell-surface bound complement regulatory activity is necessary for the in vivo survival of KDH-8 rat hepatoma. Immunology.

[10] Kinugasa N, Higashi T, Nouso K (1999). Expression of membrane cofactor protein (MCP, CD46) in human liver diseases. Br J Cancer.

[11] Spiller OB, Criado-García O, Rodríguez De Córdoba S, Morgan BP (2000). Cytokine-mediated up-regulation of CD55 and CD59 protects human hepatoma cells from complement attack. Clin Exp Immunol.

[12] Law PT, Wong N (2011). Emerging roles of microRNA in the intracellular signaling networks of hepatocellular carcinoma. J Gastroenterol Hepatol.

[13] Negrini M, Gramantieri L, Sabbioni S, Croce CM (2011). microRNA involvement in hepatocellular carcinoma. Anticancer Agents Med Chem.

[14] Ladeiro Y, Couchy G, Balabaud C (2008). MicroRNA profiling in hepatocellular tumors is associated with clinical features and oncogene/tumor suppressor gene mutations. Hepatology.

[15] Ariizumi S, Katagiri S, Katsuragawa H, Kotera Y, Yamamoto M (2007). Sectionectomy is suitable for patients with T2 hepatocellular carcinoma according to the modified International Union against Cancer TNM Classification. Dig Surg.

[16] Lu ZJ, Liu SY, Yao YQ (2011). The effect of miR-7 on behavior and global protein expression in glioma cell lines. Electrophoresis.

[17] Gao X, Gulari E, Zhou X (2004). In situ synthesis of oligonucleotide microarrays. Biopolymers.

[18] Pan W (2002). A comparative review of statistical methods for discovering differentially expressed genes in replicated microarray experiments. Bioinformatics.

[19] Treon SP, Mitsiades C, Mitsiades N (2001). Tumor cell expression of CD59 is associated with resistance to CD20 serotherapy in patients with B-cell malignancies. J Immunother.

[20] Nakagawa M, Mizuno M, Kawada M (2001). Polymorphic expression of decay-accelerating factor in human colorectal cancer. J Gastroenterol Hepatol.

[21] Simpson KL, Jones A, Norman S, Holmes CH (1997). Expression of the complement regulatory proteins decay accelerating factor (DAF, CD55), membrane cofactor protein (MCP, CD46) and CD59 in the normal human uterine cervix and in premalignant and malignant cervical disease. Am J Pathol.

[22] Junnikkala S, Jokiranta TS, Friese MA, Jarva H, Zipfel PF, Meri S (2000). Exceptional resistance of human H2 glioblastoma cells to complement-mediated killing by expression and utilization of factor H and factor H-like protein 1. J Immunol.

[23] Weichenthal M, Siemann U, Neuber K, Breitbart EW (1999). Expression of complement regulatory proteins in primary and metastatic malignant melanoma. J Cutan Pathol.

[24] Kojima A, Iwata K, Seya T (1993). Membrane cofactor protein (CD46) protects cells predominantly from alternative complement pathway-mediated C3-fragment deposition and cytolysis. J Immunol.

[25] Zell S, Geis N, Rutz R, Schultz S, Giese T, Kirschfink M (2007). Down-regulation of CD55 and CD46 expression by anti-sense phosphorothioate oligonucleotides (S-ODNs) sensitizes tumour cells to complement attack. Clin Exp Immunol.

[26] Janssen EA, Slewa A, Gudlaugsson E (2010). Biologic profiling of lymph node negative breast cancers by means of microRNA expression. Mod Pathol.

[27] Herranz H, Cohen SM (2010). MicroRNAs and gene regulatory networks: managing the impact of noise in biological systems. Genes Dev.

[28] Reinhart BJ, Slack FJ, Basson M (2000). The 21-nucleotide let-7 RNA regulates developmental timing in *Caenorhabditis elegans*. Nature.

[29] Sempere LF, Christensen M, Silahtaroglu A (2007). Altered microRNA expression confined to specific epithelial cell subpopulations in breast cancer. Cancer Res.

[30] Lan FF, Wang H, Chen YC (2011). *Hsa-let-7g* inhibits proliferation of hepatocellular carcinoma cells by downregulation of *c-Myc* and upregulation of *p16^INK4A^*. Int J Cancer.

[31] Connolly E, Melegari M, Landgraf P (2008). Elevated expression of the miR-17-92 polycistron and miR-21 in hepadnavirus-associated hepatocellular carcinoma contributes to the malignant phenotype. Am J Pathol.

[32] Yang F, Yin Y, Wang F (2010). miR-17-5p promotes migration of human hepatocellular carcinoma cells through the p38 mitogen-activated protein kinase-heat shock protein 27 pathway. Hepatology.

[33] Zheng J, Dong P, Gao S, Wang N, Yu F (2012). High expression of serum miR-17-5p associated with poor prognosis in patients with hepatocellular carcinoma. Hepatogastroenterology.

[34] Chen L, Jiang M, Yuan W, Tang H (2012). miR-17-5p as a novel prognostic marker for hepatocellular carcinoma. J Invest Surg.

